# Rats chirp with their mouth full: During an experimental meal, adult male Wistar rats emitted flat ultrasonic vocalisations upon feeding

**DOI:** 10.3389/fnbeh.2023.1089631

**Published:** 2023-02-06

**Authors:** Gaelle Champeil-Potokar, Léa Kreichati, Olivier Rampin, Isabelle Denis, Nicolas Darcel, Vincent Bombail

**Affiliations:** ^1^Physiology of Nutrition and Feeding Behaviour Unit (PNCA, UMR 0914), University of Paris-Saclay—AgroParisTech—National Research Institute for Agriculture, Food and Environment (INRAE), Paris, France; ^2^Animal Behaviour and Welfare Group, Scotland’s Rural College, Edinburgh, United Kingdom

**Keywords:** vocalisation, eating behaviour, rats, Monte Carlo analysis, food reward, behavioural sequence analysis

## Abstract

Rats produce ultrasonic vocalisation (USVs) that are classified into different types, based on their average frequency. In pups 40 kHz USVs are produced upon social isolation, and in adults USVs can be associated with affective states and specific behavioural patterns (i.e., appetitive 50 kHz vocalisations of frequency range 30−100 kHz, or aversive 20 kHz vocalisations of frequency range 18−30 kHz). Generally, USVs of frequency around 50 kHz are linked to activation of brain reward pathways, during anticipation or experience of rewarding stimuli. Previous studies have described several subtypes of 50 kHz USVs, according to their acoustic properties. We asked whether USV production might be relevant to feeding behaviour. We recorded USVs from 14-week old adult rats during the satisfaction of a physiological need: refeeding following mild food deprivation (17 h overnight fast). We analysed a 10 min consummatory phase, preceded by a 10 min anticipatory phase, as a control for the experimental meal. Following identification of USV subtypes, we applied frequentist and Bayesian (Monte Carlo shuffling) statistical analyses to investigate the relationship between USV emission and rat behaviour. We found that it was not total USV quantity that varied in response to food consumption, but the subtype of USV produced. Most importantly we found that rats who feed tend to produce flat USVs of a frequency around 40 kHz. Beyond the previous reports of circumstantial association feeding-flat USVs, our observation directly correlate vocalisation and ingestive behaviour. Our study highlights that, in addition to quantification of the production rate, study of USV subtypes might inform us further on rat consummatory behaviour. Since this vocalisation behaviour can have a communicative purpose, those findings also illustrate nutrition studies might benefit from considering the possible social dimension of feeding behaviour.

## Introduction

Rats produce ultrasonic calls (herein referred to as ultrasonic vocalisations, USVs) in response to stimuli deemed to be social and non-social (Brudzynski, 2013a; Wöhr and Schwarting, 2013; Simola and Granon, 2019). Rat pups produce long 40 kHz distress calls upon maternal separation(e.g., Boulanger-Bertolus et al., 2017). Older juvenile and adult rat USVs are sometimes used to communicate about their emotional state, in a process that has been termed ethotransmission (Brudzynski, 2013b). Twenty-two kHz USVs are associated with fear (Borta et al., 2006; Schwarting and Wöhr, 2012) and anxiogenic experiences (Vivian et al., 1994) or the sexual refractory period (Barfield and Geyer, 1972). USVs at a frequency around 50 kHz (generally between 30 and 100 kHz) are associated with positive emotional states (Burgdorf and Panksepp, 2006; Simola and Brudzynski, 2018a,b).

Over the years, USV detection in rat experimentation has proved a useful tool to assess response to appetitive experiences, such as the administration of certain drugs that possess rewarding properties, and the anticipation of their effects (Wright et al., 2010, 2012; Sadananda et al., 2012; Simola et al., 2012a,^2014^), play (Knutson et al., 1998), rat tickling (LaFollette et al., 2017), or exposure to odours associated with tickling (Bombail et al., 2019). USV production is altered during feeding-related situations, such as the anticipation of sugary food rewards (Buck et al., 2014) and food pellets (Brenes and Schwarting, 2015), and sucrose self-administration (Browning et al., 2011).

Those observations are supported by the identification of a neural substrate for 50 kHz vocalisation behaviour, since neuropharmacology studies suggest that positive 50 kHz USVs are associated with dopaminergic stimulation of the nucleus accumbens shell (Burgdorf and Panksepp, 2006; Thompson et al., 2006; Rippberger et al., 2015; Mulvihill and Brudzynski, 2019).

Amongst the 50 kHz USVs indicative of positive affective states, a distinction was initially made between flat and frequency-modulated 50 kHz calls (Burgdorf and Panksepp, 2006), based on the narrow or wide USV bandwidth, respectively. A few years later, 14 different USV subtypes were identified in rats recorded following injection of the psychostimulant amphetamine (Wright et al., 2010), the administration of which increases biogenic amine release, including striatal dopamine (Fleckenstein et al., 2007). This led to a classification made on USV shape and bandwidth on an audiogram. More recently, using an unsupervised deep learning/artificial intelligence algorithm named DeepSqueak, it is suggested there might be up to 18 subtypes of USVs (Coffey et al., 2019). Throughout this manuscript we will be referring to the classification of USV subtypes described by Wright et al. (2010). The ethological significance of this diversity in vocalisation types is still largely unknown, yet a number of recent observations are emerging.

Studies on play behaviour, using Monte Carlo shuffling analysis, reveal specific USVs might be associated with specific behaviours (Burke et al., 2017). For instance, during play anticipation, calls described as “split,” “composite,” and “multi-step” were associated with running and jumping behaviour. A total of 50 kHz trill calls were associated with anticipation of a social interaction, but not a food reward (Burke et al., 2021). Changes in USV subtypes were seen when individuals were exposed to repeated tickling sessions (Garcia et al., 2015), as frequency modulated USVs (referred to above as wide bandwidth USVs) were increased. The authors also reported that USV subtype differences might reflect differences in attributed reward value (Garcia et al., 2015). More recently Seidisarouei et al. (2021) reported that anticipation and consumption of different rewards (social or food) were associated with distinct USV production profiles. Specifically, presentation of the rewards deemed to be non-social (sucrose solutions) led to higher “flat” USVs production.

In previous experiments we had initially hypothesised USV production would be associated with food preference, and the positive response to being fed after a mild food restriction (Champeil-Potokar et al., 2021). Our initial quantitative analysis did not show an association between USV expression pattern and experimental meals. We revisited our recordings with the aim to look at how qualitative USV production and feeding behaviour were associated, in other words we asked whether specific USV subtypes were produced upon feeding.

Following the observation that rats produce USVs upon feeding, we sought to investigate this phenomenon. Since we and others have not detected quantitative differences in vocalisation during meals (Mulvihill and Brudzynski, 2018; Champeil-Potokar et al., 2021), we tested the hypothesis that specific USV subtypes might be associated with feeding behaviour. We used several complementary approaches to analyse behavioural recordings. Here, we report that rats produce USVs while chewing their food, and that the flat USV at 40 kHz are the most abundant.

## Materials and methods

### Animals

**FIGURE 1:**
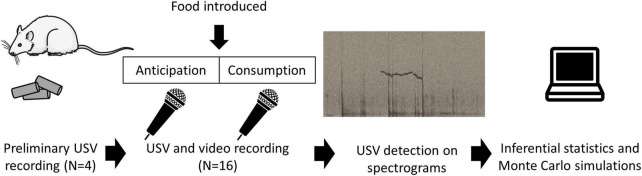
Outline of the experimental procedure. Preliminary observations on four rats who were fed their usual pellets were used to optimise recording conditions. The USV data for this study were generated using 16 other rats went through a protocol described previously (Champeil-Potokar et al., 2021). Following a mild overnight food restriction, rats were recorded during the anticipation and consumption of an experimental meal. USVs were identified from spectrograms and data were analysed using Inferential statistics and Monte Carlo simulations.

**FIGURE 2:**
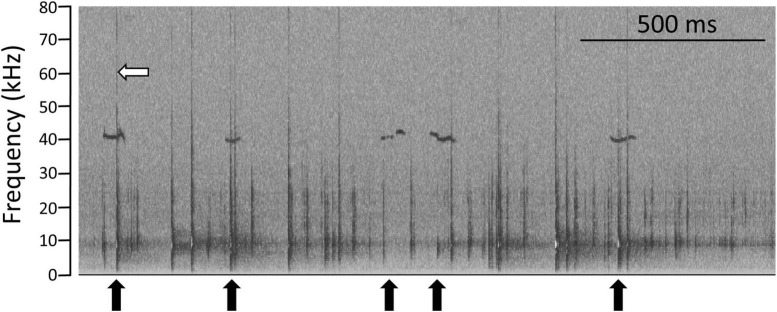
Identification of flat USVs associated with chewing food pellets. Spectrogram representing the frequency of sounds recorded with the ultrasonic microphone, while a rat was eating food pellets. When rats chewed food, the breakage of pellets produced a sound across all frequencies, in the form of the vertical lines (for example white arrow). In this illustration, rats produced flat USVs (horizontal) at around the frequency of 40 kHz, sometimes while chewing pellets (black arrows). Horizontal scale bar represents 500 milliseconds.

The experiments were carried out in accordance with the European Union directive of 22nd September 2010 (2010/63/EU) and were approved by the local ethics committee (COMETHEA) and by the French Ministry for Research (authorisation APAFIS#19571). To optimise our recording conditions (Figure 1), we carried out preliminary recordings on four male rats (3-month old), not used in the rest of this study, who were fed their usual food pellets after a mild food deprivation (described below in section “Experimental design of the test meal”). The insights from those recordings are described in the first paragraph of the section “Results.” Examples of signal obtained feature in the audiograms are presented in Figures 1, 2.

For the experiment reported herein, sixteen male Wistar rats (4 weeks old) were purchased from Janvier Labs, maintained in our animal facility, and kept in pairs in standard rodent cages under controlled light (light:dark 12:12 h reverse cycle, on at 20:00, to ensure testing during the active dark phase) and temperature conditions (22 ± 2°C). They were kept under those conditions for a 2-week acclimation period, and they were fed a standard laboratory diet (M25 Extralabo, Dietex, France) and water *ad libitum*.

Following the 2-week habituation to the laboratory after their delivery, rats were engaged in an experimental protocol for 8 weeks, consisting of food preference trials, olfactory testing, habituation to experimental food (see below), until they were recorded during the final experimental meal (Champeil-Potokar et al., 2021).

### Diets

Food pellets were custom made on site (Unité Expérimentale des Sciences de l’Animal et de l’Aliment de Jouy, INRAE) on a modified version of the AIN−93 M diet (Reeves et al., 1993) with casein as protein source, maize starch, maltodextrin and sucrose as carbohydrates and soy oil as lipids. Protein content of the diet was, respectively, 21% [of energy intake (EI)] for Normal Protein (NP) pellets (given daily during the whole experiment period), 6% EI for Low Protein (LP) pellets (test meal during USV recording) and 60% EI for High Protein (HP) pellets (test meal during USV recording). Carbohydrate was adapted to balance protein, being 61% EI in NP diet, 76% EI in LP pellets and 22% EI in HP pellets. described in detail elsewhere (Champeil-Potokar et al., 2021).

### Experimental design of the test meal

The recordings investigated for this study were from 16 rats (aged 14 weeks old) fed NP diet *ad libitum* throughout the experiment. USVs were recorded before (10 min) and during (10 min) the test meal, given after a mild fast of 17 h during the light (rest) phase, to increase the motivation to eat. During the anticipation phase food pellets were contained in a tea ball, and could therefore be detected but not consumed. We chose a 17 h fast as this allowed us to remove the food over the light phase of the cycle (typically low activity and low feeding) and test the animals the next day during the dark phase (high activity and high feeding). As the food was removed during a phase when rats feed less, we assumed this would enhance motivation to eat but would be a milder intervention than longer food restrictions that encompass the dark phase (and might induce a stronger stress response to maintain energetic balance, e.g., Nowland et al., 2011).

The test meals were carried out during the dark (active) phase, under dim red light (4 lux), in the same type of transparent polycarbonate cages used for housing (42.5 cm × 26.6 cm × 18.5 cm), covered with their metal grid lid. The rats had been habituated to handling and their experimental cage during testing sessions over 8 weeks (as per above (Champeil-Potokar et al., 2021); the same cage was used for each animal for the entire duration of the experiment. After each visit to the cage, any faeces and urine patches were removed and the shavings were mixed. Rats from the same pair residing together were tested at the same time. They were recorded in two cages separated by cardboard walls, which according to our unpublished data strongly decreased ultrasound transmission intensity between cages and prevented a microphone from clearly recording USVs from the other cage.

### USV and video recording and analysis

Ultrasonic vocalisations were recorded at a sampling rate of 384 kHz with an ultrasonic microphone (sensitivity range: 10 to 160 kHz; M500−384, Pettersson Elektronik, Sweden) attached 10 cm above the cage lid and a freeware sound-recording programme (Audacity 2.1.3).^1^ Sound events were identified from spectrograms generated in Audacity with fast Fourier transform (Hanning window of size 1024). USVs were defined as discrete sonic events of peak frequency 20−100 kHz and duration 10−150 ms, with a minimum of 20 ms between 2 events. We chose such broad criteria to be inclusive of any ultrasonic events not previously described in the literature. The current classification of 50 kHz USV subtypes sometimes lack information on frequency, we therefore recorded subtype and estimated frequency independently. For USV subtype identification, we used published USV descriptions [following amphetamine injection, as described in Wright et al. (2010)], and our own experience in detecting USVs emitted during rat tickling or play behaviour (Bombail et al., 2019; Hammond et al., 2019). When further confirmation was required, the recordings were played at 0.05 × speed to listen to the sounds in the human audible range. To visually assess USV frequency, we estimated the projected median value of the total USV trace onto the frequency axis (to the nearest multiple of 5 kHz). Audacity recordings were visually assessed by two researchers naïve to experimental conditions.

Rats were video recorded during the test meals (Sony 12.0 mega pixels HDR-XR-500 Handycam). Recordings were analysed using a freeware video player that allows frame by frame analysis at the resolution of 40 ms (64-bit PotPlayer).^2^ For each rat, the video and USV track timings were synchronised using the sound of a timer audible on both recordings, at the start of each phase. The timing of this event could be assigned to a video frame (at the resolution of 40 ms) and to the USV spectrogram (to the nearest millisecond). This was used to investigate what actions the rats were performing upon USV production. We also used scan sampling at the resolution of 1 s to describe behaviour over the 10 min of anticipation or food consumption phases (600 observations per individual and phase). The ethogram of all the behaviours detected in the recordings is described in Table 1, it follows criteria based on our previous work (Bombail et al., 2019, 2022; Hammond et al., 2019; Champeil-Potokar et al., 2021).

**TABLE 1:** Ethogram for behaviours detected in the scan sampling study of the anticipatory and consummatory meal phases (*only in the test meal phase) those behaviours are mutually exclusive.

Behaviour	Description
Up explore	Sniffing and exploring, while rearing
Down explore	Sniffing and exploring, interaction with litter, eating (litter or coprophagia)
Sniff food	Olfactory investigation of the tea ball containing the food pellets (or of the food pellets*)
Move	Locomotor behaviour (when down or rearing), along the horizontal plane
Grooming	Includes partial grooming sequences
Dig litter	Dig litter
Rearing	Rat is on his hind legs
Lying down	Lying down, with lack of perceivable movement
Immobile	Lack of perceivable movement
Feeding*	Bites food from trough, chews food

### Statistical analysis

Descriptive statistics, one-way non-parametric ANOVA and two-way ANOVA were performed using Prism 5.0 (GraphPad Software Inc., San Diego, CA, USA). Data normality was assessed using the D’Agostino-Pearson test. Further statistical analyses, a linear regression model and Monte Carlo Shuffling, were performed using R (version 3.6.3) and R-Studio (2021.09.1 + 372 “Ghost Orchid”).

We observed that the number of vocalisations by USV subtype was similar in rats given LP or HP pellets as test meal, indicating that the type of pellet had no detectable impact on USV production, then datasets from LP or HP groups were merged to carry out further analyses.

A logistic regression model was used to investigate correlations between vocalisation frequencies and co-occurrent behaviours (i.e., occurring upon USV production), taking account individual variability.

In order to investigate variation in USV production between anticipation and consumption of food, we carried out a Delta USV calculation. This was inspired by the Delta SVS calculation which was used to investigate the differences in vocalisation rates between conditions for a given individual and USV subtype (Seidisarouei et al., 2021). We calculated the Delta USV score by subtracting the number of USV emitted in the anticipation phase to the number emitted in the consumption phase, for each USV subtype and for each individual rat. We compared the Delta USV scores using the non-parametric Kruskall Wallis test.

In order to correlate USV subtypes and specific observed behaviours, we opted for co-occurrence analyses through probabilistic approaches. A Monte-Carlo shuffling analysis was performed to further assess the link between vocalisation subtypes and behaviours. This allowed us to estimate the probability that each vocalisation and behaviour co-occurred above chance levels (*p* < 0.05). This analysis accounted for individual differences in event occurrence and difference in relative rates of vocalisation among animals. A script was designed firstly to count, for a given recording session, the number of co-occurrences of each vocalisation subtype, with each of the coded behavioural categories (Table 1). Then, for recorded session (each individual), vocalisations were randomly assigned a time within the duration of the observation period (shuffling) and the number of behaviour-vocalisation co-occurrences again computed. This shuffling was repeated 10,000 times and the total number of co-occurrences of each USV subtype with each type of behaviour was tabulated. Based on the distribution of these counts, we assigned a distance (expressed in standard deviation z-score) to the actual number of occurrences. Shuffling was performed separately for each rat and all z-scores averaged to generate the final, average z-score values. We consider this probabilistic approach to be efficient as long as the observed events are not too rare (more than approximately five events observed in the whole data set amongst all individual sessions). Here we present and discuss average z-scores >2 or <−2 corresponding to a probability of 0.05 or below that co-occurrence could have occurred by chance.

## Results

### 40 kHz flat USVs can be associated with feeding behaviour (chewing food pellets)

Prior to this work, we had serendipitously detected USV production coinciding with feeding, while rats were chewing food pellets or sugar cubes (Hammond et al., 2019). In order to investigate whether this observation was reproducible and carry out preliminary tests for this study, we fed four rats with their usual food pellets. All USVs identified during and around feeding phases could be categorised according to descriptions from the literature (Wright et al., 2010). USV production over 10 min varied greatly between individuals (0.4 to 8.9 USV/min). Two individuals produced 22 kHz USVs, indicating they could be experiencing fear or anxiety. This prompted us to ensure our test animals were well habituated to handling and the experimental procedure, prior to recording them, in order to maximise vocalisations (since USV production may be inhibited by stress, e.g., Ishiyama and Brecht, 2016]. Video and audiogram analyses revealed that when food pellets were broken off by the rat’s teeth and chewed, their breakage produced brief sounds containing all measured frequencies (<1 ms long, frequency from 0 to >100 kHz, vertical lines on Figure 1). These preliminary observations indicated that during the food consumption phases, while chewing their food, rats produced flat USVs at the frequency of approximately 40 kHz (as illustrated on Figure 2). We verified this was not a recording artefact by checking that this phenomenon was only observed during a meal, and through analysing recordings of food pellets broken manually in the absence of rats, and rats recorded in the absence of food pellets. The manual breakage of a food pellets only led to the production of a sharp cracking sound, the vertical lines seen on Figure 1 (described above), and when rats were not feeding, we never observed the coincidence of flat USVs with such vertical spectrogram patterns.

### USV production and behaviour during anticipation and consumption of a meal

We identified in total 778 USVs (486 USV/10 min for LP and 292 USV/10 min for HP) during the anticipation phase and 652 USVs (333 USV/10 min for LP and 319 USV/10 min for HP) during the food consumption phase. Although there were great quantitative differences between the subtypes of USVs produced, we did not detect any significant food pellet effect [USV subtype factor *F*_(13_,_182)_ = 18.62, *p* < 0.0001; food pellet factor, *F*_(1_,_182)_ = 0.01, *p* = 0.93, interaction *F*_(13_,_182)_ = 0.35, *p* = 0.98]. Since pellet type did not affect USV production profile, we merged data from LP- and HP-fed rats, to further investigate USV production during the feeding phase.

**FIGURE 3:**
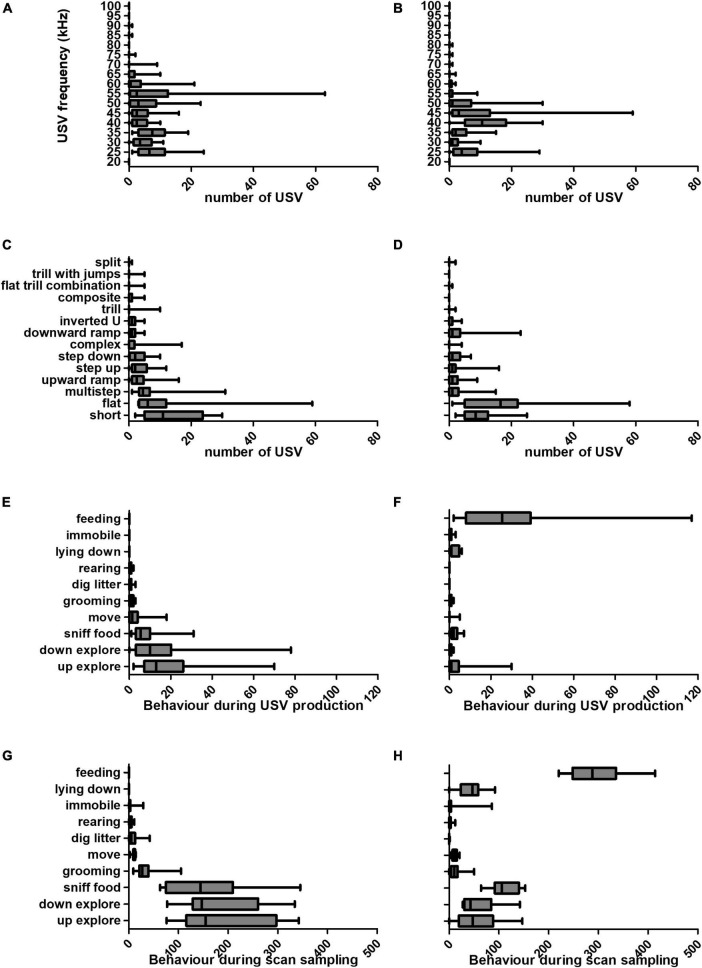
Characteristics of USVs emitted (frequency and subtype), and behaviours recorded during the anticipatory and consumption meal phases. We observed a shift in USV properties (frequency and subtype) between the anticipatory and consumption meal phases. Estimated sound frequency (in kHz) of USVs produced during the anticipatory **(A)** and consumption **(B)** meal phases. USV subtypes produced by each rat during the anticipatory **(C)** and consumption **(D)** meal phases. Number of each behaviour performed upon USV production in anticipatory **(E)** and consumption **(F)** meal phases. Number of each behaviour performed in anticipatory **(G)** and consumption **(H)** meal phases. All measures were made over the duration of the anticipation and consumption phases (10 min each, or 600 s). Graphs are box-and-whiskers plots describing the minimum value, 25th percentile, median, 75th percentile, and maximum value (*N* = 16 rats).

Over the course of the experiment, behaviour, USV frequency and subtype differed between food anticipation and consumption phases. In spite of marked individual differences, a pattern of USV production emerged. The number of USVs and associated behaviours are presented for the anticipatory and consummatory phases (Figure 3). The distribution of USV frequency was altered in presence of food (Figures 3A, B). USVs of frequency 50 kHz and above constituted 337 (43%) of total USVs produced during anticipation, and 111 (17%) during the consumption phase. In contrast, during the consumption phase, 410 USVs (63%) had frequencies between 35 and 45 kHz, whereas only 238 (31%) during anticipation.

The general linear model results are presented in Table 2. The predicted frequencies can be calculated by subtracting the estimate value for each co-occurrent behaviour from the intercept estimate. Statistical analysis using the logistical regression model revealed a significant correlation between behaviour observed upon USV production and vocalisation frequencies (*F* = 10.268; *p* < 10^–11^). USVs produced while rats were feeding were of a predicted frequency of 44 kHz (*p* = 0.016). Additionally, the predicted frequencies were 25 kHz for USVs produced during grooming (*p* = 0.00059) and 45 kHz for lying down (*p* = 0.022). It should be noted that a strong effect of each individual was highlighted by this statistical model (*F* = 12.512; *p* < 10^–15^), thus indicating a very strong heterogeneity between animals as demonstrated in Figure 3.

**TABLE 2:** Coefficients for the general linear model describing the frequency of USVs produced depending on the co-occurring behaviour (*p < 0.05, **p < 0.001).

Behaviour	Estimate	Std. error	*t*-value	*P* (>|t|)
(Intercept)	72.500	11.615	6.242	8.36e−10**
Feeding	−28.102	11.643	−2.414	0.016098*
Grooming	−47.500	13.743	−3.456	0.000588**
Immobile	6.667	6.706	0.994	0.320551
Lying down	−27.500	11.995	−2.293	0.022234*
Move	10.000	6.146	1.627	0.104259
Sniff food	−20.000	10.388	−1.925	0.054692
Up explore	−12.500	12.365	−1.011	0.312467

Figures 3C, D show USV subtypes produced during anticipatory and meal phases. The three most abundant USV subtypes during the anticipation phase were short, flat and multistep USVs (representing 63% of total USVs), and during the consumption phase, there were more short, flat and downward ramp USVs (representing 64%).

Next, we investigated the actions performed by rats while they were vocalising (Figures 3E, F), in comparison to the scan sampling data, which describe rat behaviour every second of the 10 min investigated (Figures 3G, H). During the anticipation phase 74% of USVs were produced during up- or down-explore behaviours (Figure 3E), which represented 63% of all behaviours (Figure 3G). This raises the possibility that rats produced more USVs during those behaviours because these behaviours were more frequent. Interestingly, our data shows that more USVs were produced during feeding, since while feeding represented only 49% of all recorded behaviours during the consumption phase (Figure 3F), 75% USVs emitted during this phase were associated to feeding (Figure 3H).

### Flat USVs are more frequent during the meal consumption phase

**FIGURE 4:**
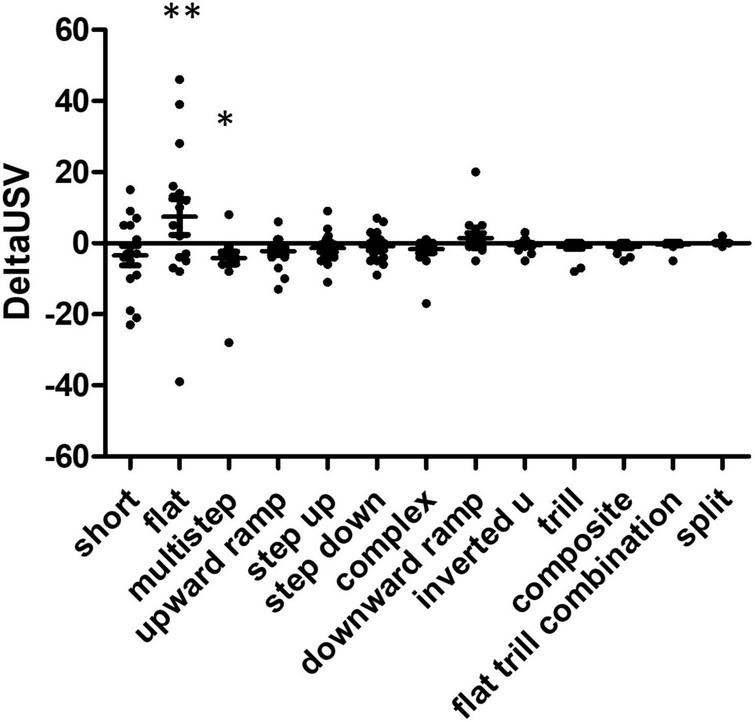
Comparison of USV production in food consumption phase reveals over-representation of specific USV subtypes. Delta USV calculations were performed for each USV subtype and each rat. Statistical analysis reveals significant differences between food anticipation and consumption phases (Kruskall Wallis *W* = 23.88, *p* = 0.0211), some USVs were over- or under-represented in the food consumption phase, compared to the anticipatory phase. The *post hoc* Dunn’s multiple comparison test allowed the identification of significantly different USV subtypes, these are indicated by asterisks on the graph: flat vs. multistep (^**^p < 0.01) and multistep vs. trill (*p < 0.05) *N* = 16 rats.

Using anticipation as the control (basal state), we calculated the delta USV for each rat during the consumption phase (Figure 4). We excluded trill with jumps USVs (five events), since those were produced by only one individual during the anticipation phase. Kruskal Wallis testing showed a difference between USV subtypes (*W* = 23.9, *p* = 0.02), and Dunn’s multiple comparison tests indicate the significant comparisons were flat vs. multistep and multistep vs. split. Overall, rats produced more flat USVs and fewer multistep USVs during the consumption phase than during the anticipation. When considering USVs that were produced while the food was being chewed (vertical audiogram signal, see above and Figure 2), 30% of total USVs (198/652) were produced while rats were chewing their food, while 43% of flats (124/287) were produced while rats were chewing their food (Chi square 1, 1261 = 6.89, *p* = 0.009). Flat USVs were more likely than other USVs to be produced while rats were chewing food.

### Monte Carlo simulation shows flat USVs are associated with feeding behaviour

**FIGURE 5:**
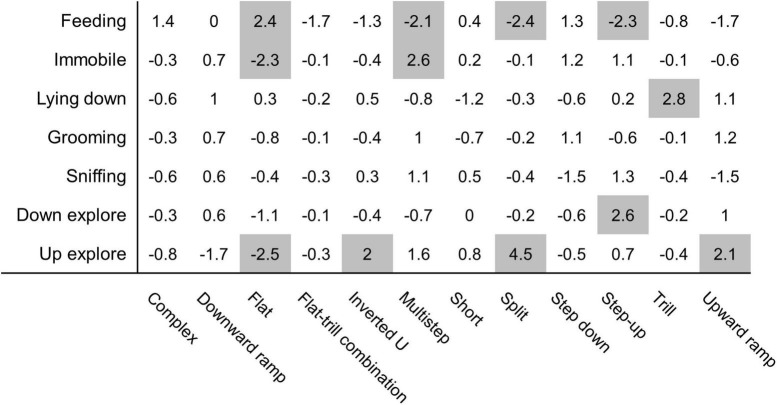
Monte Carlo shuffling analysis reveals association between USVs produced and specific behaviours. Each USV produced during the feeding phase, and the behaviour performed upon vocalising, was analysed using Monte Carlo shuffling simulations. Those analyses resulted in *z*-values representing a probability of association. A *z*-value above 2 and below −2 (highlighted in grey) represent, respectively, a statistically significant association or lack of association between behaviour and USV (*p* = 0.0455 or below) *N* = 16 rats.

Monte Carlo shuffling was used to investigate associations between behavioural categories and vocalisation subtypes. Our analysis indicates that several associations were probably not due to chance (Figure 5). Too few USVs were observed for “rearing,” “move,” and “dig litter” behaviours, and those behaviours were not included in the analysis. “Feeding” behaviour shows very particular patterns of association since it was associated with flat USVs (z-score = 2.4) but was associated with fewer multistep USVs (z-score = −2.1), split USVs (z-score = −2.4), and step-up USVs (z-score = −2.3). The “immobile” behaviour is strongly exclusive of flat USVs (z-score = −2.3) and associated with multistep USVs (z-score = + 2.6). “Lying down” behaviour is strongly associated with trill USVs (z-score = + 2.8). “Down explore” had a positive association with step up USVs (z-score = 2.6). The “up explore” behaviour was not associated with flat USVs (z-score = −2.5) and, conversely, associated with inverted U USVs (z-score = 2), split USVs (z-score = 4.5), and upward ramp USVs (z-score = 2.1). “Grooming” and “sniffing” behaviours were not associated with any subtype of USVs.

## Discussion

We sought to investigate USV production associated with feeding. Associations between rat USVs and behaviours during anticipation and meal consumption were drawn using statistical and probabilistic analysis of behavioural and vocalisation sequences. We observed that USVs emitted upon food consumption were predominantly flat shaped USVs of 40−45 kHz frequency (herein referred to as 40 kHz flat USVs). Since we observed these USVs in older individuals and in a different context, we postulate they were distinct from the pup alarm calls produced upon maternal separation (Boulanger-Bertolus et al., 2017).

In keeping with results by others (e.g., Mulvihill and Brudzynski, 2018), we find that food consumption does not affect total number of USVs produced, compared to meal anticipation (Champeil-Potokar et al., 2021); in contrast the proportion of flats and other USVs, as well as their frequencies, changes (e.g., as seen in Simola et al., 2012b). Note that since we used different inclusion criteria, for instance cut off lower frequency of 20 kHz here, instead of 30 kHz in Champeil-Potokar et al. (2021), the number of USVs detected slightly differed from our previous report.

This USV-behaviour association identified upon consumption of the experimental meal is consistent with the findings of Takahashi et al. (2010), who used a different approach. In an observational study where behaviour and USVs were investigated over several hours of home cage life, Takahashi et al. (2010) found that a cluster of 40 kHz flat USVs was associated with phases of feeding. In 24 h recordings of rats where food availability was temporally restricted, Opiol et al. (2015) also described a greater flat USV production (of unspecified frequency) when food was available. Flat USV production (of unspecified frequency) has also been described in other experimental models of ingestive behaviour (Mulvihill and Brudzynski, 2018), using ethanol and sugary cereal treats, considered to have high valence and attractiveness. Our observations indicate that similar USVs are produced for less palatable food such as usual pellets. Interestingly by comparing USV production patterns in a range of attractive stimuli, non-social situations rewards elicited more flat USVs than social interactions (Seidisarouei et al., 2021). This is consistent with our observed USV production pattern detected in up-explore behaviours, where the rat might be seeking social interaction, away from the empty cage. When exploring above them, rats produced fewer flats and more USVs with frequency modulation (higher frequency range) such as upward ramp and step up USVs. Our data confirm and expand on previous findings, as we show USVs were produced while the rats were chewing their food. We identified 40 kHz flat USV that started before and ended after the chewing of food pellets. Beyond the circumstantial association feeding-flat USVs, this links vocalisation to consummatory ingestive behaviour. In other words, rats do chirp with their mouth full.

What might be the neurophysiological correlates of feeding-associated 40 kHz flat USV production? Several lesion or neuropharmacology studies (Grant et al., 2015; Vecchia et al., 2018; Costa et al., 2020), including using inhibition of USVs by haloperidol (Thompson et al., 2006; Wright et al., 2013; Mulvihill and Brudzynski, 2019) and diazepam (de Oliveira Guaita et al., 2018) support the hypothesis that dopaminergic signalling is involved in promoting/stimulating vocalisation behaviour. Interestingly, flat USVs are the least observed USVs when rats perform the very rewarding act of playing (Burke et al., 2017). Flat USV production can be associated with a depression of the dopaminergic tone. For instance, Ciucci et al. (2009) show FM USVs are replaced by flats in rats after reducing dopaminergic signalling with haloperidol or 6-hydroxydopamine administration. Similarly rats with mutations in the Cacna1c gene, putatively suffering from altered dopaminergic signalling (Terrillion et al., 2017) and associated with mood disorders, produce more flat USVs (Sangarapillai et al., 2022). However, others have shown flat USVs might be more represented in conditions of hyperdopaminergic activity (Wright et al., 2010; Simola et al., 2012b; Simola and Costa, 2018). It is therefore possible the production of our 40 kHz flat USVs represents a modulation (higher or lower) of dopaminergic tone upon chewing the food.

We found no significant impact of meal pellet components (HP vs. LP) on USV production. This could be because the mild (17 h in the light phase, when rats rest) food restriction administered to increase motivation to eat made all food attractive and rewarding, regardless of meal type. The question of food palatability/preference and USV production, and their interaction with physiological status (fed/food-restricted) or social context, requires further investigations. In accordance with our previous results (Champeil-Potokar et al., 2021), there was a great degree of individual variability in USV response, as we also found in rat playful handling or tickling, a form of heterospecific play where the human experimenter simulates rough and tumble play with a rat (e.g., Bombail et al., 2021). This variability in USV production has been previously linked to the diversity in emotional behaviour and laboratory animal temperament between individuals (Sundarakrishnan and Clarke, 2022). Discrimination in total USV production has been used to investigate rats with low and high positive affect, links between chronic stress and response to amphetamine administration (Kõiv et al., 2016), anxiety/depressive traits (Mällo et al., 2007, 2009). Interestingly these characteristics are linked to DA signalling (Buck et al., 2014). Extremely high or low USV production is also linked to response sensitivity to amphetamine treatment (Kaniuga et al., 2016). Rats who produce more USVs upon tickling exhibit higher optimism, cognitive bias toward positive outcomes (Rygula et al., 2012). USV production also differs between goal- vs. sign-tracker rats in Pavlovian learning tasks, where the salience of a conditioned stimulus is linked to varying USV production (Sangarapillai et al., 2021). One important question would be to investigate possible links between individual USV production patterns and feeding behaviour.

The fact that food intake might be accompanied by emission of 40 kHz flat USVs (in addition to other sensory cues) begs the question of the impact, if any, the USVs might have on feeding behaviour of a listener rat. Playback studies would be interesting, to investigate those might be referential calls (Manser and Manser, 2016). In rats, flat USVs have a role in social communication (Brudzynski, 2013b) and the fact they might not always be associated with pleasurable or social stimuli (Burgdorf et al., 2008; Wöhr et al., 2008) suggests one of their functions might be a role in social behaviour coordination (Burgdorf et al., 2008). Food associated calls are found in other species, their meaning remains elusive (Clay et al., 2012). In some cases, speculations on their role point toward social signalling. For instance, in white-faced capuchin monkeys, *Cebus capucinus*, feeding-associated calls might reduce aggression, through advertising food ownership (Gros-Louis, 2004). Conversely, food call may also advertise the presence of food for others to share resource location, and therefore vocalisations can be part of collaborative behaviour (O’Bryan et al., 2021). It has been shown that observation of a rat eating a choice of two food types influences food choice and food intake in an observer (Galef and Wigmore, 1983). This phenomenon, the “information-centre” hypothesis, constitutes an example of social transmission of diet preference in rats. This transmission of dietary information was found to be independent of whether the rats were long domesticated, hungry, unfamiliar to the observer, young, and took place for solid food and liquids (Galef and Wigmore, 1983). It has been shown this transmission might take place partly through chemosensory cues, including gustatory contact (Laland and Plotkin, 1993). But in a rat world rich in odours and sounds (Burn, 2008), olfaction is not the sole mechanism of food information transmission (Galef, 1990), the congruence of sensory modalities might constitute a stronger message. Feeding-associated USVs could therefore play a role in socially guided food choices.

In conclusion, our study demonstrates that specific (40 kHz flat) USVs are emitted by rats during a meal, notably during chewing, with a great variation between individuals. This variability, together with the difficulty in identifying USVs, might constitute challenges, although new tools (e.g., Coffey et al., 2019) should allow us to overcome these limitations. The meaning of these USVs remains to be investigated and could reflect hedonic manifestation and/or social calls. Our findings, in line with previous published results, shed light on the fact that nutritional studies should include description of rat vocalisation behaviour when they eat. Future experiments will involve comparing the USV response to meals with a range of hedonic value and should take into account the individual variations in physiology and taste preference. Future recordings of rat USVs in relation to feeding behaviour should also take into account naturally occurring variations in pro-social behaviour among individuals. Food intake is a process that has a social dimension. After decades of feeding single caged rats in nutrition research, the time might have come to listen to their conversations. This might give us something to talk about at dinner tonight.

## Data availability statement

The raw data supporting the conclusions of this article will be made available by the authors, without undue reservation.

## Ethics statement

This animal study was reviewed and approved by the experiments were carried out in accordance with the European Union directive of 22nd September 2010 (2010/63/EU) and were approved by the Local Ethics Committee (COMETHEA) and by the French Ministry for Research (authorisation APAFIS#19571).

## Author contributions

GC-P: methodology, investigation, and writing—reviewing and editing. LK: investigation and writing—reviewing and editing. OR: methodology and writing—reviewing and editing. ID: conceptualisation, methodology, validation, funding acquisition, and writing—reviewing and editing. ND: conceptualisation, methodology, formal analysis, writing—original draft, and writing—reviewing and editing. VB: supervision, project administration, funding acquisition, conceptualisation, methodology, formal analysis, writing—original draft, and writing–reviewing and editing. All authors contributed to the article and approved the submitted version.

## Footnotes

1
www.audacityteam.org

2
potplayer.daum.net
